# Diagnostic and progression biomarkers in cerebrospinal fluid of Alzheimer’s disease patients

**DOI:** 10.1186/s12916-024-03270-w

**Published:** 2024-02-08

**Authors:** Miyo K. Chatanaka, Ioannis Prassas, Eleftherios P. Diamandis

**Affiliations:** 1 Department of Laboratory Medicine and Pathobiology, University of Toronto, Toronto, ON Canada; 2 Laboratory Medicine Program, University Health Network, Toronto, ON Canada; 3 Lunenfeld-Tanenbaum Research Institute, Mount Sinai Hospital, Toronto, ON Canada

**Keywords:** Alzheimer’s disease, Biomarker, Non-invasive, SMOC1, Neuropentraxin, Diagnostic test, Reference range

## Abstract

In this commentary, we address a paper published by Johnson et al. by assessing the robustness of their method to discover diagnostic biomarkers in Alzheimer’s disease (AD). In addition, we examine how these newly discovered and previously discovered biomarkers, can play a role in assisting patients with AD and those at risk for developing AD, with an emphasis on the translational hurdles that accompany such discoveries.

## Introduction

Recently, Johnson et al. and the Dominantly Inherited Alzheimer Network (DIAN) published an excellent paper describing the discovery of new diagnostic and progression biomarkers for Alzheimer’s disease (AD) in cerebrospinal fluid (CSF) of patients with mutant or wild-type genes, namely amyloid precursor protein (APP), Presenilin 1 (PSEN1), and Presenilin 2 (PSEN2) [1]. They identified SPARC-related modular calcium-binding protein 1 (SMOC1) and another 33 proteins that are significantly altered (increased or decreased during the disease course) in autosomal dominant Alzheimer disease (ADAD) mutation carriers, in comparison to non-carriers. Using these biomarkers, they correctly categorized carriers from non-carriers across the disease time course and compared their data with current and emerging Aβ, phosphorylated tau (pTau), and other biomarkers. These new, single, or composite biomarkers, whose concentration is changing over many years (sometimes 40 years earlier than clinical AD manifestation) have the potential to be used for both sporadic (common) or inherited (very rare) AD risk stratification, in efforts to prevent, or slow down progression by using current and emerging therapies. The same group has recently published additional data by analyzing 1305 proteins in brain tissue, CSF, and plasma from patients with sporadic AD, triggering receptor expressed on myeloid cells 2 (*TREM2*) risk variant carriers, patients with ADAD, and healthy individuals and they identified 8 brain, 40 CSF, and 9 plasma proteins that were altered in individuals with sporadic AD [2]. In this commentary we wish to address two issues: 1. To assess the robustness of their biomarker discovery strategy described in [1] by comparing their new biomarkers with already reported predictive biomarkers in CSF of late-onset AD (LOAD); 2. To examine how these newly discovered and additional biomarkers could be used to help AD patients or those at risk for developing AD in the future, with emphasis on potential translational hurdles.

The authors of the ADAD paper [1] assembled a group (*N* = 59) of promising progression AD biomarkers based on prior findings in LOAD patients described in multiple studies, including our own [3–9].

Of these, 33 were informative (Fig. 2) and were measured once in CSF of ADAD patients (carriers and non-carriers) cross-sectionally, by using selected reaction monitoring (SRM) mass spectrometry. By calculating the approximate timing of ADAD clinical manifestations, and using molecular family data, they modeled biomarker changes before and after clinical disease manifestation in both carriers and non-carriers. Some plots of biomarker changes over time, in relation to the approximate calculated timing of disease onset are presented in the paper (their Fig. 1).

In our own papers with a similar objective (3–5; not referenced by Johnson et al.), we assembled a list of 30 CSF proteins by selecting brain-specific proteins, using Protein Atlas, that were also present in one, or both, CSF proteomes from apparently normal individuals and LOAD [4]. Our detailed rationale and findings are described in our cited papers [3–5]. We then measured the 30 selected proteins by SRM mass spectrometry in CSFs from cognitively normal, mild cognitive impairment (MCI), moderate, and severe LOAD [5]. Among the proteins that were altered, 5 proteins were common between the study of Johnson et al. and ours. We confirmed these to be proteins whose concentration changes with LOAD progression from normal, to MCI, to moderate, and to severe AD. As expected, these five proteins belonged to the fifth category of proteins (as categorized by Johnson et al.) which coincide with the onset of brain atrophy and are decreased in CSF of LOAD. These included neuronal and neurosecretory proteins such as VGF, neuropentraxin, and its receptor (NPTX and NPTXR, respectively), suggesting considerable synaptic and neuronal loss. The decreases of CSF NPTXR in LOAD were confirmed by multiple methods and were found to correlate with amyloid load and PET findings [7–9].

We concluded that the discovery strategies between the Johnson et al. and our own studies provide partially similar, overlapping data, even if the patient samples used are very different (LOAD vs ADAD) and come from different biobanks. However, due to the relatively small number of the selected proteins for SRM quantification in the two studies (< 100), we are almost certain that additional proteins in CSF, which change concentration as the disease progresses, likely exist, if it is considered that the CSF proteome contains at least 3000 proteins [3, 4]. The newer work of Johnson et al. [2] confirmed this suggestion.

## Clinical applicability of the new candidate biomarkers

A non-invasive biomarker test that can detect any disease, (including AD) years before clinical presentation is valuable, because it can contribute to the better understanding of disease pathogenesis and natural history, allow physicians to deliver earlier therapies, or used for disease prevention. Critical performance characteristics of candidate biomarkers include sensitivity (ability of the biomarker to detect disease), specificity (the biomarker should be negative in non-affected individuals), non-invasiveness, and low cost. As we stressed elsewhere, for screening asymptomatic individuals (such as patients with prodromal AD), the positive and negative predictive value of the test should be high (usually > 90%) to confidently rule-in or rule-out disease presence [10, 11]. The sensitivity and specificity of a test can be adjusted by selecting appropriate cut-offs. But tests that are applied to screening of asymptomatic individuals also have important limitations, some being the increased lifelong patient anxiety [10], false positive and false negative results. Also, analyzing CSF as a screening biofluid would be problematic, since its collection is invasive, especially if collected longitudinally at multiple points over time. The suggestion of starting therapy of true positive patients very early (e.g., at adolescence), and continuing it for life will likely reveal potentially serious side effects. The relatively recent therapy of AD with amyloid-removing antibodies was plagued by brain swelling, with 3 reported fatalities [12]. Cost is another factor, currently estimated around $26,000 per year [12]. Fortunately, there are a few examples of the feasibility of life-long pharmacological interventions for disease prevention (such as use of statins for preventing atherosclerosis) and a similar strategy may be used to slow down or halt AD progression.

For every diagnostic test, the patient’s result is compared with results derived from a reference (apparently healthy, non-affected) population. For SMOC1 (Fig. 1c), and the other proposed protein biomarkers in Ref 1, the reference population for LOAD will be almost impossible to reliably identify, due to contamination of the group of non-affected individuals with asymptomatic patients with early/prodromal AD. In other words, when deriving reference ranges (RR) that will be used to identify LOAD, a percentage of the “reference” population (given that the prevalence of prodromal or mild LOAD is high) will have prodromal but asymptomatic disease. The large inter-individual variability of SMOC1 between patients and individuals labeled as controls will lead to very wide and likely difficult to use RR due to the high rates of false positive and more frequently, false negative results. The calculated upper limit of normal will be set at an inappropriately high level. For example, SMOC1 values differ by about fourfold in non-carriers, about 15 years earlier than disease manifestation (data calculated from Fig. 1c). Similar comments apply to other time points and to ADAD patients (data not shown). Nevertheless, some studies such as the ones sponsored by the DIAN do collect CSF that can be used to establish reference ranges. Such studies could remove preclinical AD individuals based on other good biomarkers such as amyloid beta and p-tau, but this task is difficult and not error-free.

In Table 1, we summarize important analytical and clinical obstacles for using SMOC1-like biomarker testing to identify prodromal phases of chronic diseases, such as AD.

**Table 1:** Challenges for implementing diagnostic and predictive biomarkers for early diagnosis, therapy, and prevention of Alzheimer’s disease

Analytical and logistic challenges	Clinical challenges
Collection of CSF, once or repeatedly, is invasive	Life-long therapy of AD may be necessary
Test must demonstrate excellent analytical sensitivity and specificity	Side effects of pharmacological or other interventions should be minimal and not life-threatening
Test cost should be acceptable to society	Tests must display excellent clinical sensitivity and specificity, along with high positive and negative predictive value. False positive and false negative results should be minimal, and a confirmatory test is necessary to resolve ambiguous results
	It may be challenging to derive reliable reference ranges for the biomarkers of interest due to difficulty in finding true non-diseased individuals and including patients with prodromal and asymptomatic disease. Reference ranges may need to be derived for various age groups, since the identified biomarkers change non-linearly and non-unidirectionally over time. Reference ranges may be highly wide due to inter-individual variation

## Concluding remarks

Johnson et al. used a unique patient cohort to identify a handful of CSF proteins whose concentration is changing continuously and in a non-linear and non-unidirectional fashion, years before, and after clinical disease manifestation. As shown in the paper [1] some proteins increase in early stages of the disease and later decrease over time. The non-linear and non-unidirectional changes in the informative CSF proteins introduce another complicating parameter when trying to derive RR since these, must be derived for various time-points during disease progression (a very difficult feat). Some of these proteins were also identified by us and others, using LOAD patients. Despite the interesting findings of Johnson et al. [1], important hurdles of translating this exciting data in LOAD, either for disease prevention or for instituting earlier, and likely life-long therapies, whose efficacy and safety are currently unknown.

## Data Availability

Not applicable.

## Abbreviations

AD: Alzheimer’s disease
ADAD: Autosomal dominant Alzheimer disease
APP: Amyloid precursor protein
CSF: Cerebrospinal fluid
DIAN: Dominantly Inherited Alzheimer Network
LOAD: Late-onset AD
MCI: Nild cognitive impairment
NPTX: Neuropentraxin
NPTXR: Neuropentraxin receptor
PSEN1: Presenilin 1
PSEN2: Presenilin 2
pTau: Phosphorylated tau
RR: Reference ranges
SMOC1: SPARC-related modular calcium-binding protein 1
SRM: Selected reaction monitoring
*TREM2*: Triggering receptor expressed on myeloid cells 2
